# Recovery Dynamics of Photosynthetic Performance and Antioxidant Defense in Resurrection Plants *Ramonda serbica* and *Ramonda nathaliae* After Freezing-Induced Desiccation

**DOI:** 10.3390/plants14172760

**Published:** 2025-09-03

**Authors:** Bekim Gashi, Fitim Kastrati, Gergana Mihailova, Katya Georgieva, Eva Popova, Erzë Çoçaj, Kimete Lluga-Rizani, Qëndrim Ramshaj

**Affiliations:** 1 Laboratory of Plant Physiology, Department of Biology, Faculty of Mathematics and Natural Sciences, University of Prishtina, Eqrem Çabej Street No. 51, 10000 Prishtina, Kosovo; fitimkastrati25@gmail.com (F.K.); erze.cocaj@uni-pr.edu (E.Ç.); 2 Institute of Plant Physiology and Genetics, Bulgarian Academy of Sciences, Acad. G. Bonchev Str., Bl. 21, 1113 Sofia, Bulgaria; gmihailova@bio21.bas.bg (G.M.); georgieva.katya.m@gmail.com (K.G.); evapopova68@gmail.com (E.P.); 3 Department of Biology, Faculty of Mathematics and Natural Sciences, University of Prishtina, Eqrem Çabej Street No. 51, 10000 Prishtina, Kosovo; kimete.lluga@uni-pr.edu (K.L.-R.); qendrim.ramshaj@uni-pr.edu (Q.R.)

**Keywords:** drought stress, Gesneriaceae, low temperatures, non-enzymatic antioxidants, photosynthesis, rehydration, stress-induced proteins

## Abstract

Resurrection plants such as *Ramonda serbica* and *Ramonda nathaliae* are gaining scientific attention due to their exceptional ability to withstand extreme drought and cold. This study is the first to evaluate the changes in photosynthetic activity, antioxidant defense, and the role of protective proteins during the early hours of recovery of these species after freezing-induced desiccation. Specimens collected from natural habitats where temperatures dropped below −10 °C were rehydrated under controlled conditions, and measurements were taken at multiple time points from 1 h up to 7 days after recovery. Both species demonstrated a gradual increase in photosynthesis, with the CO_2_ assimilation rate significantly improving after 24 h and reaching full restoration by day 7. This recovery aligned with increases in relative water content and stomatal conductance. Photosystem II efficiency was fully restored within 72 h. Notably, *R. nathaliae* exhibited higher thermal dissipation during stress than *R. serbica*. Antioxidant activity peaked between 1 and 3 h of rehydration and returned to baseline by day 7. Additionally, early rehydration stages triggered the accumulation of stress-related proteins such as dehydrins, early light-inducible proteins, small heat shock proteins, and fatty acid amide hydrolase. These results provide valuable insights into the desiccation–rehydration mechanisms of *Ramonda* species, demonstrating that they fully recover physiological functions within seven days and highlighting species-specific stress responses during early rehydration.

## 1. Introduction

Extreme environmental conditions, including both low temperatures and drought, are among the most important factors that affect plant growth and development, and can also limit their distribution and productivity [1]. Climate change has extended dry periods during summer and extreme temperatures during winter in some areas [2]. Therefore, both drought and low positive and negative temperatures are stress factors for most non-tolerant plant species. Temperatures around 0 °C (cold stress) mainly affect basic physiological processes in plants, such as photosynthesis, sugar metabolism, electron transfer reactions, and protein synthesis [3,4]. However, subzero temperatures (freezing stress) can lead to cell dehydration or ice formation in the apoplast and can even cause damage to cellular compartments and tissue death [5]. The impact of these temperatures depends on plant species. Temperate species acclimatize during cold exposure to ensure freezing tolerance, whereas tropical and subtropical plants remain extremely sensitive even to mild cold [6,7]. Therefore, for the survival of perennials and resilient annuals, frost resistance is essential, relying on the ability of each plant to acclimate to low temperatures [8]. Consequently, the acclimatization period in fall is crucial for starting physiological changes that allow plants to survive freezing temperatures during winter.

Resurrection plants survive desiccation to an air-dried state and quickly restore normal physiological function upon rehydration [9]. The European resurrection angiosperm species, *Haberlea rhodopensis* Friv., *Ramonda nathaliae* Pančić & Petrović, *Ramonda serbica* Pančić and *Ramonda myconi* (L.) Rchb are capable of surviving both drought in summer as well as sub-zero temperatures in winter [10,11,12,13]. From these species, only *R. myconi* grows in the Iberian Peninsula, while all other species grow in the Balkan Peninsula [14,15,16,17]. They belong to the Gesneriaceae family and, unlike other tropical and subtropical gesneriads, these species have also developed mechanisms to tolerate subzero temperatures during winter. Furthermore, considering that *R. serbica* and *R. nathaliae* grow in their natural habitats at temperatures around 20–30 °C during summer [10], their ability to withstand frosts during winter and revive from these conditions becomes very important for research. Freezing tolerance in terms of photosynthetic adaptation was reported in *H. rhodopensis* [12,18], *R. myconi* [14], and in *R. serbica* and *R. nathaliae* [10,11]. *R. serbica* and *R. nathaliae* grow in different countries in the Balkans. The distribution of *R. nathaliae* populations mainly extends to North Macedonia, Kosovo, Serbia and Greece, while *R. serbica* mainly in Albania, Kosovo, Serbia, North Macedonia, Greece and Bulgaria [15,19,20]. These two species grow in natural environments with different conditions, *R. serbica* under cooler and more humid conditions, while *R. nathaliae* in harsher conditions during the summer, drier and with higher temperatures, and is considered more resistant to these conditions than *R. serbica* [21].

Tolerance to drought and extreme temperatures is a complex process that involves changes at the metabolic, molecular, biochemical, physiological, and morphological levels [22,23,24]. Furthermore, to survive these conditions, plants must overcome two main types of stress that prevail, oxidative and mechanical stress. In general, resurrection plants overcome the mechanical stress resulting from cell shrinkage through dehydration by cell wall folding and vacuole compartmentalization, which positively affect the maintenance of cellular integrity [25,26]. On the other hand, during dehydration, various metabolic disorders occur, that increase cellular oxidative stress, leading to the accumulation of reactive oxygen species (ROS). Resurrection plants have evolved two main mechanisms to balance the creation and elimination of ROS: poikilochlorophylly (chlorophyll degradation and dismantling photosynthetic apparatus) and homoiochlorophylly (preservation of most of the chlorophyll and photosynthetic apparatus) [24]. *R. serbica* and *R. nathaliae* belong to the homoiochlorophyllous group, as they retain their photosynthetic apparatus during dehydration both in summer and at sub-zero temperatures [10,11,21].

Photosynthesis is considered one of the most sensitive processes to water deficit resulting from both drought and frost [10,11,17]. During drying, the reduction in photosynthetic activity leads to a disruption in the balance between energy capture and energy utilization, thus leading to ROS production [27,28]. These excess molecules react with cellular macromolecules, causing oxidative damage and disruption of cellular functions. However, ROS also function as crucial signaling molecules, activating plant defense mechanisms [22,29]. These protective mechanisms for ROS scavenging are much more effective in resurrection plants by the activation of enzymatic (peroxidase (POD), superoxide dismutase (SOD), catalase (CAT), glutathione reductase (GR)) and non-enzymatic (carotenoids, ascorbate, tocopherols, glutathione) antioxidant defenses [30,31,32,33,34]. Furthermore, an increase in antioxidant defense during low temperature stress under ex situ conditions has been previously reported in *R. nathaliae*, *R. serbica* and *H. rhodopensis* [10,30]. In resurrection plants, the oxidative stress at subcellular level is alleviated by the synthesis of numerous protective compounds such as sugars and various proteins [25]. Freezing-induced desiccation of *R. nathaliae*, *R. serbica* and *H. rhodopensis* was ensured by the accumulation of dehydrins and early-light inducible proteins (ELIPs) [11,12].

A characteristic feature of resurrection plants is downregulation of photosynthetic activity as a primary line of defense against freezing and drought stress, which is accompanied by increased thermal energy dissipation [24]. During cold acclimation and freezing-induced desiccation, resurrection plants such as *H. rhodopensis*, *R. nathaliae*, *R. serbica*, and *R. myconi* exhibit xanthophyll cycle de-epoxidation, modifications of photosynthetic proteins, and chlorophyll–protein rearrangements [10,11,12,13,35]. Homoiochlorophyllous species have also been shown to maintain high stability of thylakoid pigment–protein complexes under these conditions [10,36,37]. Cold stress reduces key photosynthetic proteins (D1, D2, PsaA/B, cytochrome *b*_6_*f*) in *H. rhodopensis* [12] and increases the abundance of some LHC proteins in *R. nathaliae* and *R. serbica* [10]. It has been found that frost stress significantly reduces CO_2_ assimilation rate in *R. nathaliae*, *R. myconi*, and *R. serbica*, primarily due to stomatal closure and decreased photochemical efficiency [10,14]. Following freezing-induced desiccation, full recovery of photosynthetic function occurs in *H. rhodopensis* and in both *Ramonda* species from the Balkan Peninsula after rehydration [10,35].

Although numerous studies have explored the physiological and biochemical defense strategies employed by resurrection plants under drought, low-temperature, and freezing conditions, the recovery mechanisms in resurrection plants following freezing-induced stress are less studied in general, and unexplored in *R. nathaliae* and *R. serbica*. Since these two species grow in different natural environments [15], we hypothesized that the ecological differences will affect their recovery processes after freezing, especially in the early stages, which are particularly vulnerable and important for their ability to restore their normal physiological function [35]. Therefore, the aim of this study was to investigate and compare for the first time the changes in photosynthetic activity, antioxidant defense and the role of protective proteins during the early hours of recovery of two resurrection plants *R. nathaliae* and *R. serbica* exposed to freezing stress under natural conditions. It should be noted that all previous studies on freezing stress in resurrection plants have been conducted under controlled or ex situ conditions. Additionally, by comparing data on recovery after drought [15,38,39,40,41,42] and freezing stress, we aimed to identify the common features that confer tolerance to *R. nathaliae* and *R. serbica*.

## 2. Results

### 2.1. Recovery of Relative Water Content

**Figure 1:**
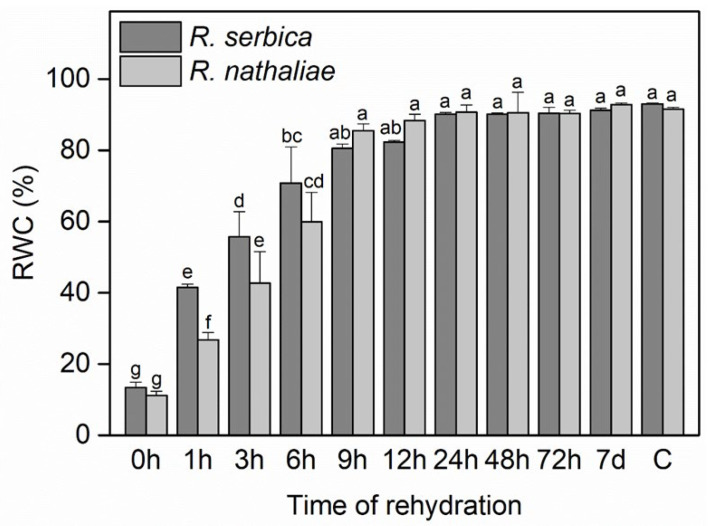
Relative water content (RWC) in leaves of *R. serbica* and *R. nathaliae* after freezing stress (0 h), and at various time points following rehydration from freezing-induced desiccation (1, 3, 6, 9, 12, 24, 48, and 78 h, and 7 days), as well as in control plants (C). The values are presented as mean ± SE, *n* = 5. Identical letters in the graph indicate no significant differences among time points following rehydration after freezing-induced desiccation, compared with the control, for both species, as determined by Duncan’s multiple range test following one-way ANOVA at the 5% significance level (*p* ≤ 0.05).

RWC dropped to around 13% in *R. serbica* leaves and approximately 11% in *R. nathaliae* leaves during the freezing period (0 h), resulting in freezing-induced desiccation (Figure 1). After the first hour of rehydration (1 h), the RWC increased to about 41% in *R. serbica* and 26% in *R. nathaliae*. This trend continued with significant increases at subsequent time points (3, 6, 9, and 12 h), and by 24 h, RWC values reached approximately 90%. Interestingly, *R. serbica* exhibited significantly higher RWC values after the first three hours of rehydration compared to *R. nathaliae*. However, by the sixth hour (6 h), the RWC values between the two species became approximately equal.

### 2.2. Photosynthetic Activity During Stress and Recovery

**Figure 2:**
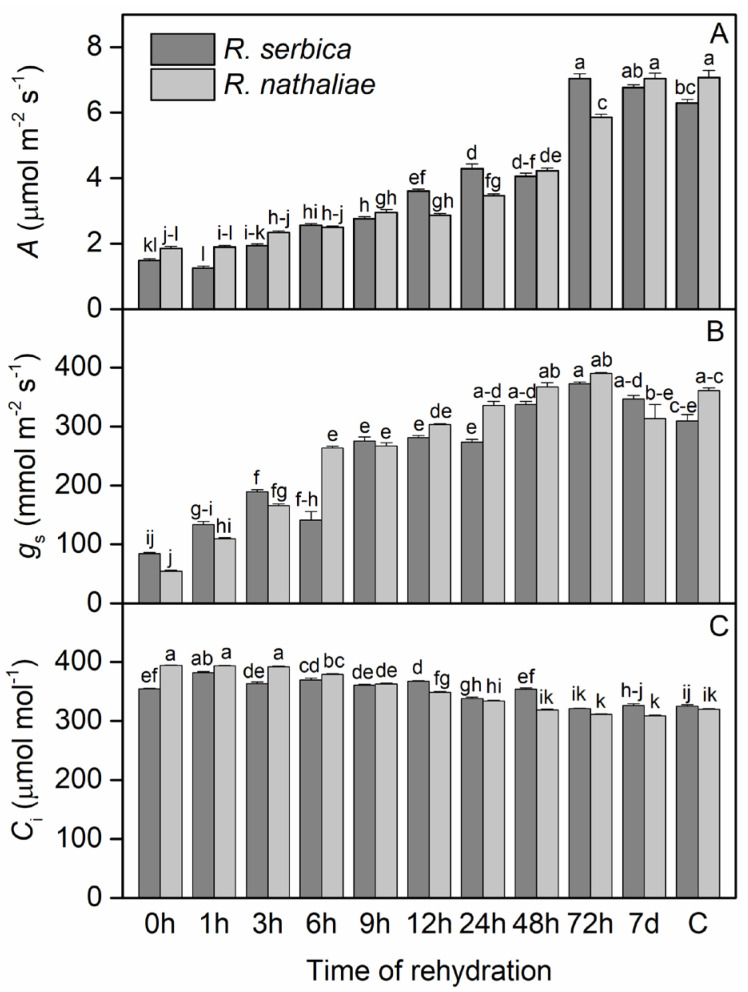
Changes in CO_2_ assimilation rate (*A*; (**A**)), stomatal conductance (*g_s_*; (**B**)) and sub-stomatal CO_2_ (*C_i_*; (**C**)) concentration in leaves of *R. serbica* and *R. nathaliae* after freezing stress (0 h), and at various time points following rehydration from freezing-induced desiccation (1, 3, 6, 9, 12, 24, 48, and 78 h, and 7 days), as well as in control plants (C). The values are presented as mean ± SE, *n* = 9. Identical letters in the graph indicate no significant differences among time points following rehydration after freezing-induced desiccation, compared with the control, for both species, as determined by Duncan’s multiple range test following one-way ANOVA at the 5% significance level (*p* ≤ 0.05).

The photosynthetic rate (*A*) of *R. serbica* and *R. nathaliae* after freezing stress, during early recovery, and in control plants showed significant differences between species, particularly after 24 h of rehydration (Figure 2A). During the freezing conditions that induced the severe desiccation (0 h), *A* showed a minimum value of 1.48 μmol CO_2_ m^−2^ s^−1^ in the leaves of *R. serbica* and 1.86 μmol CO_2_ m^−2^ s^−1^ in the leaves of *R. nathaliae*, compared to the control values (C), which were 6.29 and 7.07 CO_2_ m^−2^ s^−1^ respectively. The rate of CO_2_ assimilation did not change significantly in any of the species after the first hour (1 h) of rehydration (Figure 2A). It then slowly increased during rehydration up to 9 h, followed by a more significant increase after 24 h, 48 h, and 72 h of rehydration. Maximal and control-like values were fully restored by the seventh day (7 d) of rehydration, reaching 6.77 and 7.00 μmol CO_2_ m^−2^ s^−1^ in *R. serbica* and *R. nathaliae*, respectively. Overall, CO_2_ assimilation values during the first 12 h of recovery remained lower than those in the subsequent period.

Regarding stomatal conductance (*g_s_*), after the freezing period (0 h), its values dropped to a minimum in both species: 84.45 mmol m^−2^ s^−1^ in *R. serbica* and 54.82 mmol m^−2^ s^−1^ in *R. nathaliae* (Figure 2B). In contrast, sub-stomatal CO_2_ concentration (*C_i_*) reached its maximum during this period, with values of 354 μmol mol^−1^ in *R. serbica* leaves and 394 μmol mol^−1^ in *R. nathaliae* (Figure 2C). Following rehydration, *g_s_* began to increase after the first hour (1 h) in both species, with a more pronounced rise by the third hour (3 h) of recovery (Figure 2B). This upward trend continued throughout the rehydration process, reaching control levels (C) after 24 h (24 h). As for *C_i_*, our results indicate a gradual decline in both species over time during rehydration. This decrease becomes especially evident after 12 h (12 h, Figure 2C), with *C_i_* values approaching control levels after 72 h of rehydration (72 h, Figure 2C).

### 2.3. Photochemical Efficiency and Energy Dispersion During Stress and Recovery

**Figure 3:**
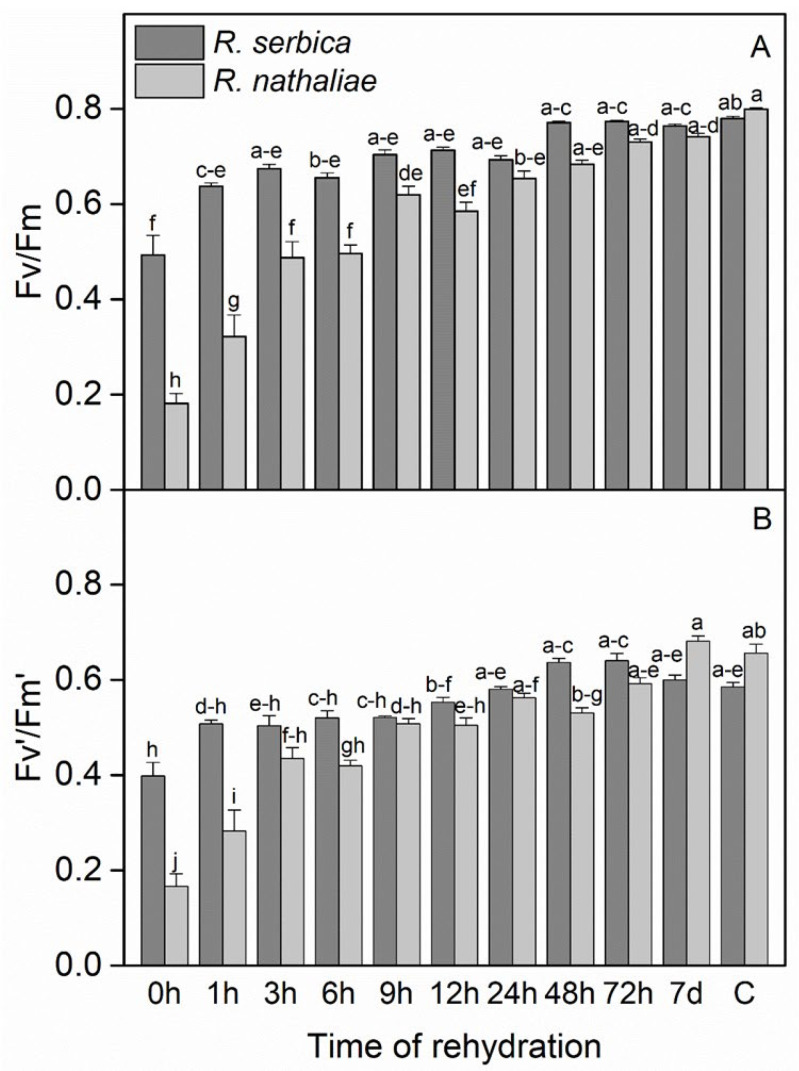
Maximum photochemical efficiency of PSII (F_v_/F_m_, (**A**)) and photochemical efficiency of PSII in the light-adapted state (F_v_′/F_m_′, (**B**)) in leaves of *R. serbica* and *R. nathaliae* after freezing stress (0 h), and at various time points following rehydration from freezing-induced desiccation (1, 3, 6, 9, 12, 24, 48, and 78 h, and 7 days), as well as in control plants (C). The values are presented as mean ± SE, *n* = 9. Identical letters in the graph indicate no significant differences among time points following rehydration after freezing-induced desiccation, compared with the control, for both species, as determined by Duncan’s multiple range test following one-way ANOVA at the 5% significance level (*p* ≤ 0.05).

The maximum photochemical efficiency of PS II (F_v_/F_m_) and the photochemical efficiency of PSII in the light-adapted state (F_v_′/F_m_′) during the early stages of recovery from freezing stress, as well as in control plants of *R. serbica* and *R. nathaliae*, showed significant differences between species, particularly during the first 1–12 h of rehydration (Figure 3). After freezing-temperature stress, F_v_/F_m_ values (Figure 3A) decreased significantly in *R. nathaliae* (0.18), while they showed a marked reduction in *R. serbica*, though to a lesser extent (0.49). The recovery of F_v_/F_m_ was gradual and closely associated with the increase in RWC (Figure 1 and 3A, respectively), as RWC increased, F_v_/F_m_ also improved.

The initial increase in F_v_/F_m_ occurred rapidly within the first hour (1 h) after rehydration, and this improvement continued progressively, becoming significantly more pronounced after 24 h (24 h) of rehydration (Figure 3A). Notably, the recovery was more marked in *R. serbica*, with a stronger statistical significance compared to *R. nathaliae* up to 6 h of rehydration. Both species reached control-like F_v_/F_m_ values of approximately 0.8 after 72 h (72 h) of rehydration (Figure 3A), indicating near-complete recovery. Similar results were observed for F_v_′/F_m_′, where after freezing stress, the minimum values reached 0.17 in *R. nathaliae* and 0.40 in *R. serbica* (0 h, Figure 3B). However, the recovery of F_v_′/F_m_′ was also rapid. After nearly 24 h (24 h) of rehydration, both species exhibited significant recovery, reaching values similar to those of the control plants (C). Specifically, F_v_′/F_m_′ values were 0.59 for *R. nathaliae* and 0.66 for *R. serbica*, indicating a fast restoration of photochemical efficiency.

**Figure 4:**
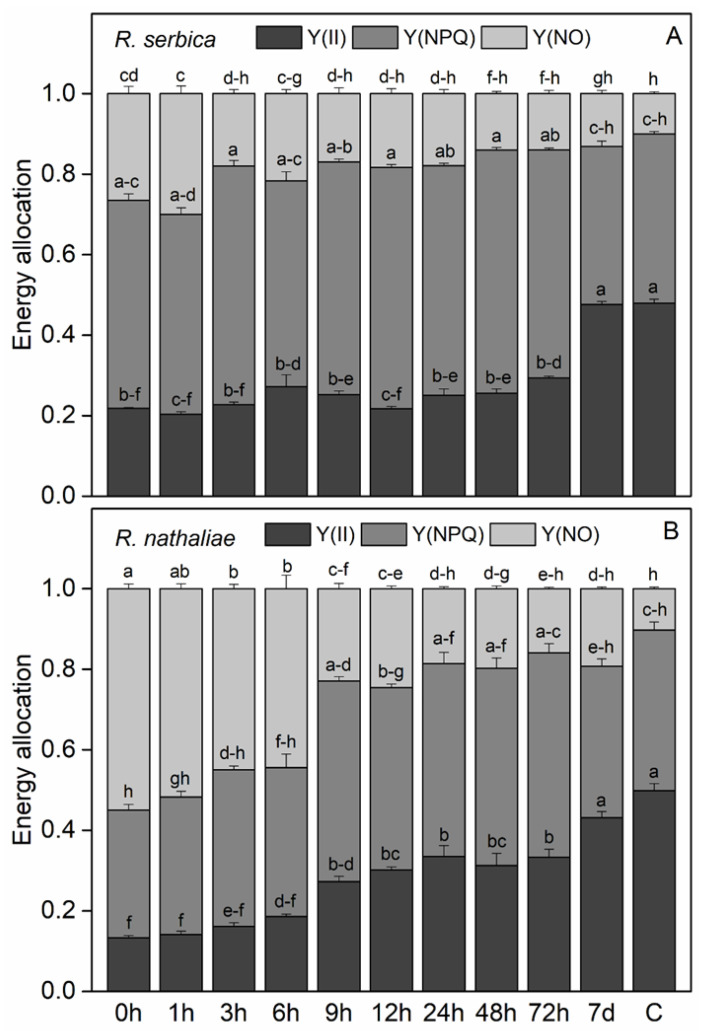
Changes in the efficiency of photochemical energy conversion in PSII under light (Y(II)), non-regulated energy dissipation quantum yield (Y(NO)) and the quantum efficiency of regulated non-photochemical quenching (Y(NPQ)) in leaves of *R. serbica* (**A**) and *R. nathaliae* (**B**) after freezing stress (0 h), and at various time points following rehydration from freezing-induced desiccation (1, 3, 6, 9, 12, 24, 48, and 78 h, and 7 days), as well as in control plants (C). The values are presented as mean ± SE, *n* = 9. Identical letters in the graph indicate no significant differences among time points following rehydration after freezing-induced desiccation, compared with the control, for both species, as determined by Duncan’s multiple range test following one-way ANOVA at the 5% significance level (*p* ≤ 0.05).

The efficiency of photochemical energy conversion in PSII under light (Y(II)) showed a significant decrease after freezing-induced desiccation, especially in *R. nathaliae* (Figure 4A and 4B, respectively). After rehydration, Y(II) did not show any significant increase during the first few hours. The efficiency of PSII gradually increased in the course of rehydration, reaching values close to the control after one week (7 d).

The low quantum efficiency of PSII during the first hours of rehydration was accompanied by high quenching of excess excitation energy (Figure 4A and 4B, respectively).Regardless some enhancement in the quantum yield of non-regulated energy dissipation Y(NO) at the freezing stage and after 1 h of rehydration of *R. serbica*, the main part of excitation energy was dissipated as regulated non-photochemical quenching NPQ, indicating its important role in avoiding photoinhibition (Figure 4A). While the values of Y(NO) gradually decreased during rehydration, those of Y(NPQ) remained high up to 72 h of rehydration. In contrast, the main mechanism of dissipation of excess energy at the freezing stage and the first 6th h of rehydration of *R. nathaliae* was through Y(NO) (Figure 4B). With further rehydration of this plant species, Y(NO) decreased and Y(NPQ) increased, but both quenching parameters reached control level after 7d of rehydration.

### 2.4. Antioxidant Responses During Stress and Recovery

The results for total phenols (TP) and total flavonoids (TF) indicate that their levels in *R. serbica* and *R. nathaliae* were about 3 times higher after freezing stress compared to the control plants (C) (Table 1). Their content further increased after the first and third hours (1 and 3 h) of rehydration, but began to decline after the sixth hour (6 h), with a gradual decrease at each subsequent stage. By the seventh day (7 d), the levels were close to those observed in the control plants (C). Similar results were obtained for antioxidant capacity, determined by the phosphomolybdate (TAC) and ferric reducing antioxidant power (FRAP) assays, as well as free radical scavenging capacity measured by DPPH and ABTS^•+^ assays (Table 1). In this context, both species exhibited markedly higher antioxidant capacity, especially in the early phases of rehydration (1, 3 and 6 h) compared to the control plants, which gradually declined after further rehydration, reaching values similar to the control plants after the seventh day (7 d). This was particularly pronounced for FRAP and for the specie *R. nthaliae* in all phases of rehydration. Similar results were obtained for the changes in DPPH and ABTS^•+^, indicating a significantly higher free radical scavenging capacity after freezing stress (0 h) and following the first and third hours of rehydration (1 and 3 h) compared to the control plants (C) (Table 1). It is worth noting that DPPH had higher values after freezing stress and at the beginning of rehydration in *R. serbica*, while ABTS^•+^ was higher in *R. nathaliae*.

### 2.5. Protein Changes During Stress and Recovery

**Figure 5:**
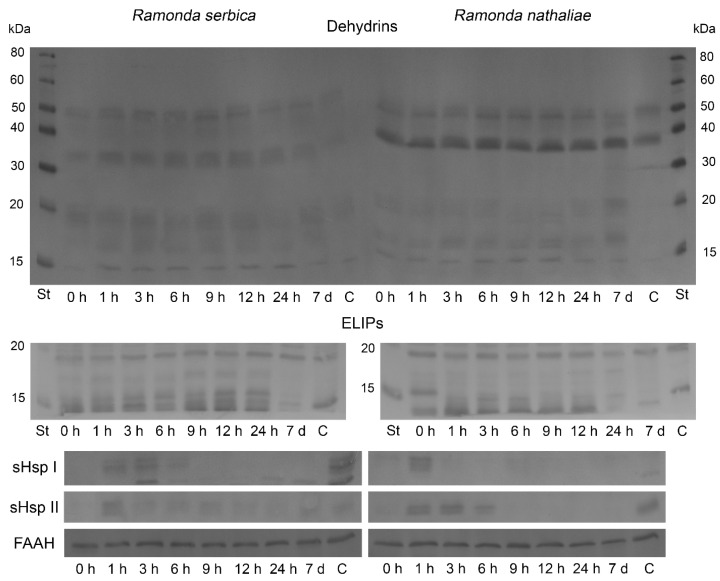
Western blot of dehydrins, ELIPs, sHsp Class I, sHsp Class II and FAAH in leaves of *R. serbica* (**left**) and *R. nathaliae* (**right**) after freezing stress (0 h), and at various time points following rehydration from freezing-induced desiccation (1, 3, 6, 9, 12 and 24 h, and 7 days), as well as in control plants (C).

The changes in the abundance of dehydrins, ELIPs, cytosolic sHsp Class I, cytosolic sHsp Class II, and fatty acid amide hydrolase (FAAH) during rehydration from freezing-induced desiccation of *R. serbica* and *R. nathaliae* were monitored by Western blot (Figure 5). Slight differences were observed in the patterns of dehydrin accumulation between *R. serbica* and *R. nathaliae*. In *R. serbica* leaves, dehydrin bands with apparent molecular weights of 50, 34, 20, 17–18, and 15 kDa were detected. In *R. nathaliae*, the bands appeared around 50, 40, 20, 17, and 15 kDa. In leaves of control *R. serbica* plants, faint dehydrin bands were detectable. Rehydration up to 12 h of dry *R. serbica* plants increased the abundance of dehydrins to some extent. After 24 h and full recovery of plants (7 d), their content decreased. In control *R. nathaliae* leaves, the dehydrin bands around 50 and 40 kDa were present only. The abundance of dehydrin proteins in *R. nathaliae* remained largely unchanged during the rehydration of desiccated plants. A slight decline in low-molecular weight dehydrins (20–15 kDa) was observed after 9 to 24 h of rehydration.

Several immunosignals of the Chl *a*/*b* binding proteins, ELIPs, within the 13–19 kDa molecular weight range were detected in both plants (Figure 5). ELIPs were detected in all analyzed samples, including control plants, but rehydration increased their content slightly up to 24 h and 12 h after recovery of plants, *R. serbica* and *R. nathaliae*, respectively. Subsequent rehydration led to a decline in the abundance of ELIPs as some isoforms of the proteins disappeared. In the controls of both *Ramonda* plants, their content was the lowest.

Western blot analysis showed the presence of the cytosolic sHsp 17.6 Class I during the first 6 h of rehydration of *R. serbica* (Figure 5). The protein was not detected in desiccated plants (0 h), but was present in controls (C). In *R. nathaliae* leaves, the sHsp Class I signal was only observed after 1 h of recovery. The protein was missing in desiccated leaves (0 h), and very faint signals could be detected in all other rehydrated samples.

Immunoblot analysis with cytosolic anti-sHsp 17.7 Class II antibody showed the presence of the protein only after 1 h of rehydration of *R. serbica* (Figure 5). sHsp Class II was not visible in desiccated plants, and very faint signals could be observed in all other rehydrated samples, including the control (C). In *R. nathaliae* plants, sHsp Class II was detected only after 1, 3, and 6 h after recovery, as well as in control plants. A faint band was present in air-dried samples (0 h).

The protein abundance of FAAH, an enzyme catabolizing N-acylethanolamines (NAEs), was enhanced during the early hours of rehydration of both *Ramonda* species (Figure 5). In *R. serbica*, after an initial slight decline in FAAH content, the protein accumulated after 3 h of recovery, and its levels remained higher compared to desiccated plants until the end of rehydration. On the other hand, FAAH content began to increase immediately after the start of rehydration in *R. nathaliae* leaves, and its abundance also remained high throughout the recovery process. At the end of rehydration (7 d), its content decreased by 25%.

## 3. Discussion

### 3.1. Dynamics of Relative Water Content

The early hours of rehydration of *R. nathaliae* and *R. serbica* following freezing-induced desiccation remained largely unexplored until now. Relative water content (RWC) is crucial for assessing recovery from dehydration and physiological restoration in resurrection plants [10,11,14,21,43]. In our study, winter temperatures in the natural habitats of both species were negative during January and February 2025, with several consecutive days below −10 °C from late January to early February. During the freezing phase (0 h), RWC was markedly reduced to approximately 10% in both *R. serbica* and *R. nathaliae* (Figure 1). These results aligned with previous findings on these two species regarding their adaptation during cold acclimation and freezing stress, where similar RWC values were observed [10]. During rehydration, *R. serbica* demonstrated significantly faster rehydration than *R. nathaliae* during the early hours (1–6 h), but differences became negligible after 9 h (Figure 1). This initial difference may be attributed to *R. serbica* greater frost resistance [10] and its larger biomass compared to *R. nathaliae* [21], factors that could contribute to a faster recovery. In contrast, our previous research during summer drought stress revealed that *R. nathaliae* demonstrated greater resistance and faster recovery than *R. serbica* [15], reflecting the different climatic adaptations of the two species. *R. nathaliae* populations are typically found in sub-Mediterranean climatic conditions, while *R. serbica* populations are adapted to continental climates with lower winter temperatures. Similarly to *H. rhodopensis* [32,35], full recovery of RWC in both *Ramonda* species was achieved by the seventh day (7 d), with RWC values comparable to those of the control samples (C) (Figure 1).

### 3.2. Alterations in Photosynthesis and Chlorophyll Fluorescence Under Stress and During Recovery

Although the lowest assimilation rate of CO_2_ (*A*) (Figure 2A) was observed in both *Ramonda* species after freezing-induced desiccation, photosynthesis remained active. We found that the recovery of CO_2_ assimilation rate was slow at the beginning of rehydration and significant enhancement was observed 24–72 h after rehydration. The lower CO_2_ uptake during the first 12 h may be attributed to the limited stomatal conductance during the early phase of recovery, which directly influences CO_2_ uptake and photosynthetic rate and efficiency. Similar findings were reported in previous studies on *R. serbica* and *R. myconi* [10,14], and this could indicate a higher level of frost resistance, adaptive capacity and fast recovery upon rehydration. The reduction in photosynthetic activity during the desiccated state is crucial, as it minimizes the production of ROS and helps preserve the structure and function of the photosynthetic apparatus [44]. Furthermore, a strong association between the decline of *A* and RWC has been observed in *R. myconi* plants [14], *H. rhodopensis* [36] and *Barbecina purpurea* [45].

The lower values of stomatal conductance after freezing stress were accompanied with enhanced sub-stomatal CO_2_ concentration, showing an inverse relationship between these two physiological parameters in *R. serbica* and *R. nathaliae* (Figure 2B and Figure 2C, respectively). This inverse relationship occurs because under desiccation, the stomata close to prevent water loss, leading to a sharp decrease in *g_s_*. Consequently, the reduction in CO_2_ assimilation rate causes an accumulation of CO_2_ in the intercellular spaces, resulting in elevated *C_i_*. Increased sub-stomatal CO_2_ concentrations under cold stress have been shown to activate photoprotective mechanisms in some resurrection plants, which helps minimize photooxidative damage and enhances their freezing tolerance [46]. Our results are also consistent with previous findings of a pronounced reduction in stomatal conductance in *Ramonda* species exposed to temperatures as low as −10 °C under ex situ conditions [11]. After rehydration, stomatal conductance slowly increased during the first 6 h in both species, reaching near-control levels after 24 h of recovery (Figure 2B). This pattern strongly correlates with the increase in RWC, suggesting that an RWC of 50% may represent a physiological threshold that allows recovery of CO_2_ assimilation. Previous studies on resurrection plants have shown that photosynthetic capacity is fully restored following rehydration. Upon rehydration, photosynthetic recovery is associated with increased *g_s_* and restored Rubisco activity [10,47,48].

Under freezing stress, the pronounced decrease in F_v_/F_m_ values in both *Ramonda* species resulted in photoinhibition of PSII, more pronounced in *R. nathaliae* compared to *R. serbica* (Figure 3). These differences may be attributed to the distinct climatic conditions in which the two species naturally occur, as previously discussed. Therefore, the more pronounced decline in photochemical efficiency observed in *R. nathaliae* under freezing stress may represent a protective mechanism to prevent overexcitation of the photosynthetic apparatus, highlighting this species comparatively lower frost tolerance relative to *R. serbica.* This result is in agreement with our earlier research on the cold and freezing stress adaptations of these plants [10]. It has been reported that cold stress reduces F_v_/F_m_, with freezing temperatures causing a stronger decline in *Ramonda* species. Drought-induced dehydration also lowers F_v_/F_m_, particularly below 50% relative water content, while downregulating photosynthesis helps overwintering plants minimize winter damage [11,13,49,50]. Tissue freezing during winter caused a significantly reduction of F_v_/F_m_ in *R. myconi*, which is linked to zeaxanthin formation; however, F_v_/F_m_ rapidly recovered and was fully restored within six hours upon warming [13].

Regarding the fast restoration of F_v_/F_m_ values of both *Ramonda* species (Figure 3A), similar results following freezing stress have been reported in other European Gesneriads. In *H. rhodopensis*, the recovery of F_v_/F_m_ was observed after 24 h of rehydration [36], while in *R. myconi*, full restoration occurred within 6 h [13].

Freezing-induced desiccation led to a notable decline in PSII photochemical efficiency, particularly in *R. nathaliae* (Figure 4). Similarly to CO_2_ assimilation rate, Y(II) remained low during early rehydration, showing no immediate improvement. However, PSII efficiency gradually recovered over time, reaching near-control levels by day seven, indicating a successful restoration of photosynthetic function in both species. Slow recovery of photochemical activity of PSII during the early stages of rehydration has also been observed in *H. rhodopensis*. This correlates with lower RWC values and high excitation pressure, prompting plants to reduce antenna size or increase heat dissipation to restore energy balance [36].

The substantial differences between the two species in energy dissipation, especially with the higher thermal energy dissipation in *R. nathaliae* (Figure 4), may be due to the different climatic conditions in their natural habitats. As mentioned earlier, *R. nathaliae,* adapted to sub-Mediterranean habitats, dissipates excess energy as heat to protect itself against photoinhibition and oxidative stress during winter, as populations growing under high-radiation show especially efficient thermal energy dissipation [10,11].

### 3.3. Antioxidant Defense Mechanisms in Response to Stress and Recovery

One of the most important protective mechanisms during freezing stress, particularly in the early stages following rehydration, is the antioxidant defense system. Consequently, the activation of antioxidant responses during both stress and recovery phases plays a crucial role in maintaining cellular redox balance and minimizing oxidative damage in resurrection plants [10]. Our results show that total phenols (TP) and total flavonoids (TF) levels in *R. serbica* and *R. nathaliae* increased significantly during the early stages of rehydration (1 and 3 h), compared to both control and freezing-stressed plants. Similarly, antioxidant activity and flavonoid levels markedly increased upon severe desiccation of *H. rhodopensis* and remained elevated after rehydration, indicating sustained defense [31,51]. Elevated flavonoid levels during freezing stress have also been reported in *H. rhodopensis* [30] and both *Ramonda* species [10]. Although research on phenolic compounds in rehydrating plants remains limited, it is well established that their accumulation in resurrection plants is a key protective strategy, helping to prevent photoinhibition, particularly during early rehydration when water influx can damage cells. The enhanced synthesis of polyphenols at low temperatures in *H. rhodopensis* leaves has also been described as a protective strategy in this species [30]. Additionally, enhanced accumulation of phenolic acids in *R. serbica* has been shown to positively affect the preservation of membrane structure during dehydration [43]. The elevated accumulation of total phenolics under stress conditions plays a vital role in scavenging free radicals, thereby aiding plants in reducing oxidative damage [52].

It was found that both *R. serbica* and *R. nathaliae* exhibited significantly elevated antioxidant capacity during the early phases of rehydration (1, 3, and 6 h) compared to control plants (Table 1), indicating its crucial role in maintaining cellular redox balance and minimizing oxidative damage. The high values of antioxidant capacity during rehydration seem to have a direct relationship with the recovery of physiological functions, especially that of photosynthesis in these two species, which serves to mitigate ROS-induced damage. Furthermore, the high antioxidant activity of the resurrection plant *Xerophyta viscosa* during the early stages of rehydration is believed to play a crucial role in survival under water stress [53]. Similarly, DPPH and ABTS^•+^ assay results showed a progressive decrease in antiradical scavenging activity with the advancement of rehydration time. This may also be related to the production of ROS, which can be much higher during the early phases of rehydration and decreases with the restoration of RWC. It has been shown that high antioxidant activity is crucial for combating stress caused by frost, as well as for the revival of *H. rhodopensis* plants [18,30].

### 3.4. The Role of Protective Proteins During Stress and Recovery

Cumulation of proteins is a common response of resurrection plants to minimize the detrimental effect of ROS and protect cell macromolecules through the dehydration-rehydration cycle [18,25,35].

Dehydrins are hydrophilic proteins belonging to LEA2 group of proteins. They prevent aggregation and stabilize the proteins in plant cells during desiccation [54]. Dehydrins accumulated in response to drought and low temperatures in resurrection plant species [11,18,55,56,57]. Our results showed different expression profiles of dehydrins in *R. serbica* and *R. nathaliae* during recovery after freezing-induced desiccation, which reflected the differences in their natural conditions. We detected similar protein patterns during desiccation of both plants at low temperatures [10,11]. It had been shown that the expression of LEA genes depended on the climatic conditions during desiccation [58]. Dehydrins maintained high abundance during rehydration in both *Ramonda* species, especially the low molecular weight dehydrins (20–15 kDa), which indicated their important role in the recovery process [35]. In control plants, these proteins were not detected. Rehydration of *H. rhodopensis* from drought- and freezing-induced desiccation showed that the low molecular weight dehydrins (22–20 and 12 kDa) were localized in the chloroplast [35].

ELIPs belong to the LHC superfamily and have photoprotective functions on the photosynthetic machinery, both under drought and freezing temperatures [59,60,61]. Western blot analysis showed the enhanced abundance of ELIPs in the process of rehydration of *R. serbica* and *R. nathaliae* after freezing-induced desiccation. This enhancement coincided with the increased levels of NPQ in both *Ramonda* species. It was reported that ELIP genes were among the most abundant transcripts during the dehydration-rehydration cycle of resurrection plants [35,58,62,63]. The restoration of PSII photochemistry was accompanied by a decrease in the ELIPs content after 7 d and 24 h rehydration of *R. serbica* and *R. nathaliae* plants, respectively. Similarly to dehydrins, we observed different patterns of accumulation of ELIPs during recovery of both *Ramonda* species and this is likely related to the different environmental conditions of their natural habitats. Previous studies showed that resurrection plants from *Gesneriaceae* family had different expression profiles of ELIPs during cold acclimation and freezing-induced desiccation [10,11].

Together with dehydrins and ELIPs, we investigated the content of the cytosolic sHsp Class I and II chaperones in the leaves of *R. serbica* and *R. nathaliae* during rehydration from freezing-induced desiccation. Plants possess a large number of over 30 different sHsp proteins [64]. Several studies report on the accumulation of sHsp in resurrection plants under drought and high temperature stresses and their importance for acquiring desiccation tolerance. Moreover, some of the sHsps are constitutively expressed in the leaves of resurrection plants, as well as in the roots [31,55,62,65,66,67,68]. Our results showed that the content of sHsp was elevated during the first hours of rehydration of *R. serbica* and *R. nathaliae*. We can suggest that sHsp Class I plays a more important role in the recovery process of *R. serbica*, being induced up to 6 h after the start of rehydration compared to *R. natahaliae*, in which this chaperone is only detected in the leaves after 1 h of rehydration. And vice versa, sHsp Class II plays a more important role during rehydration of *R. nathaliae*, being induced again up to 6 h after recovery and during the first hour after rehydration only in *R. serbica*.

FAAH is an enzyme that terminates the signaling pathway of N-acylethanolamines NAEs, which occur from polyunsaturated fatty acids [69] and is connected with ABA metabolism [70]. It was demonstrated that elevated levels of endogenous NAE 12:0 and ABA correlate with enhanced transcript content for ABA-responsive genes, coding proteins related to desiccation tolerance, including dehydrins, and lead to inhibition of seed growth in *A. thaliana* [70]. Our results demonstrated elevated protein abundance of FAAH after 3 h and 1 h of rehydration of *R. serbica* and *R. nathaliae*, respectively. We suggest that FAAH rapidly terminates the NAEs signal functions, leading to a shift in the metabolism towards recovery of both *Ramonda* species. It has been shown that similar genes are associated with desiccation tolerance of resurrection plants and seeds [71,72]. It is believed that since there is no difference in genomic organization in resurrection angiosperm species, they evolved their desiccation tolerance from the existing one in seeds [58,73].

## 4. Materials and Methods

### 4.1. Habitat Description, Sampling, and Experiment Setup

In this research, we used two species of European *Gesneriaceae* plants, *R. nathaliae* and *R. serbica*. The study was conducted on natural populations of *R. serbica* in Koprivnik (Bjeshkët e Nemuna; (42°37′47.3″ N, 20°16′01.0″ E)) and *R. nathaliae* in Glloboçicë (42°10′45.0″ N, 21°10′59.0″ E), both in Kosovo. As for *R. nathaliae*, it has a very limited distribution in Kosovo, occurring only in two populations in the Sharr Mountains (Glloboçicë and Gotovushë), growing at altitudes of about 900 m and in drier environments [19,74,75]. In contrast, *R. serbica* has a broader distribution, with several populations in the Sharr Mountains and the Bjeshkët e Nemuna, growing at altitudes ranging from 530 to 1700 m and in more humid habitats [13,19]. Regarding climatic conditions, Kosovo has a continental climate with sub-Mediterranean influences, characterized by cold winters and warm summers [76]. In this region, the lowest winter temperatures can reach as low as −20 °C in mountainous areas, while summer temperatures can rise up to 35 °C in the lowlands. In the area where the populations of *R. nathaliae* occur, there is a clear sub-Mediterranean climatic influence, while the populations of *R. serbica* experience stronger continental climatic conditions [77].

This research was carried out on plants and plant samples collected in the aforementioned locations during the first week of February 2025. During this period, minimum temperatures had reached below −10 °C for several consecutive days. During the expeditions, at least 15 leaves from different plant individuals of both species were collected and promptly frozen and preserved in liquid nitrogen to prevent degradation for subsequent analysis of secondary metabolites, antioxidant activity, and Western blot. Photosynthesis and chlorophyll fluorescence parameters (described below) were also measured during these field trips (0 h). Plants were later transferred to controlled growth conditions, at light intensity of 200 µmol m^−2^ s^−1^, temperatures ranging from 23 °C to 25 °C, and relative air humidity of 80%. In natural habitats, plants had entered a state of anabiosis due to freezing stress. After collection from their natural habitat, the plants were immediately transferred to controlled conditions (as described above), and recovery in both species was initiated by spraying and watering. Subsequently, Measurements for all the investigated parameters were taken at 1, 3, 6, 9, 12, 24, 48, 78 h, and on the 7th day after the recovery of both species in the vegetative room. For the control samples (C), all measurements were conducted in October, when meteorological conditions were considered optimal for the growth of these plant species.

### 4.2. Determination of Relative Water Content (RWC)

At each measurement stage, we assessed the relative water content (RWC) in leaves of both *Ramonda* species from control plants (C), during freezing stress (0 h), and throughout recovery (1, 3, 6, 9, 12, 24, 48, 72 h, and on the 7th day). To determine RWC, we first measured the fresh weight (FW), then the turgid weight (TW) was determined by soaking the leaves in distilled water for 24 h under laboratory conditions, and finally the dry weight (DW) (leaves were oven-dried for 24 h at 105 °C). The RWC was calculated using the formula: RWC (%) = (FW − DW)/(TW − DW) × 100 [21,78].

### 4.3. Quantification of Total Phenolic Content, Flavonoid Content, and Antioxidant Activity

The analysis of total phenolic and flavonoid contents, as well as antioxidant activity, was conducted on the leaves of both *R. nathaliae* and *R. serbica*, which were of approximately the same age and size. Leaves were collected from control plants (C) and those subjected to freezing stress (0 h), as well as at various time points during recovery: 1, 3, 6, 9, 12, 24, 48, and 72 h, and on day 7. The extraction followed [10], using 100 mg of dried leaves homogenized with 80% methanol. The resulting extract was then used for further analyses. For all the following spectrophotometric measurements, the Thermo Scientific BioMate 3S (Waltham, MA, USA), a compact UV-Vis spectrophotometer with a 190–1100 nm range, dual-beam optics, pre-configured assays, and temperature control, was used.

Total phenolic content (TP) was determined using the Folin–Ciocalteu reagent (Merck, Darmstadt, Germany) method with Na_2_CO_3_, as described [79], with minor modifications [10]. For each sample, 50 µL of methanolic extract was combined with 100 µL of Folin–Ciocalteu reagent (previously diluted with water 1:5 (*v*/*v*)) and allowed to react for 3 min, after which 100 µL 0.7 M L^−1^ of sodium carbonate (Merck, Darmstadt, Germany) was added. Absorbance was measured spectrophotometrically at 765 nm. TP concentrations were calculated based on a gallic acid standard (Merck, Hohenbrunn, Germany) curve (0–30 mg mL^−1^) and are reported as mg gallic acid equivalents per g dry weight (mg GAE/g DW).

Total flavonoid content (TF) measurement was conducted using a spectrophotometric method based on the protocol of [80], with minor adjustments as previously optimized for these plant species [10]. Specifically, 50 µL of the methanolic extract was mixed with 500 µL of water, followed by the addition of 100 µL of 5% NaNO_2_ (Merck, Darmstadt, Germany). The mixture was incubated at 50 °C for 5 min. Subsequently, 30 μL of 10% AlCl_3_ (Merck, Darmstadt, Germany) was added, and the mixture was allowed to stand for an additional 5 min. Finally, 200 μL of 1 M Na_2_CO_3_ and 240 μL of distilled water were added to the reaction mixture. Absorbance was measured at 510 nm against a blank prepared in the same manner, with the extract replaced by distilled water. TF concentrations were calculated from a catechin standard (Merck, Darmstadt, Germany) curve (0–20 mg mL^−1^) and expressed as mg catechin equivalents per g dry weight (mg CE/g DW).

Ferric reducing antioxidant power (FRAP) was conducted using the colorimetric method described [81]. A 0.1 mL methanolic extract was mixed with 1 mL sodium phosphate buffer and 1 mL of 1% K_3_[Fe(CN)_6_] (Merck, Darmstadt, Germany), then incubated at 50 °C for 20 min. Following a 10 min incubation at room temperature, the intensity of the blue-green color was measured spectrophotometrically at 700 nm. A standard curve was prepared using ascorbic acid (Sigma-Aldrich, St. Luis, MO, USA) at concentrations ranging from 1 to 2000 μM. FRAP of the samples was expressed as micromoles of ascorbic acid equivalents per gram of dry weight (μmol AAE/g DW).

DPPH radical scavenging activity was assessed using a modified version of the colorimetric method as described [82] with slight modification, 0.01 mL of the methanolic extract was adjusted to 3 mL with 80% methanol. The mixture was then combined with 2 mL of 0.25 mM DPPH solution (Sigma-Aldrich, St. Luis, MO, USA), thoroughly vortexed, and left to incubate in the dark for 30 min. Absorbance at 517 nm was used to quantify antioxidant activity, with a Trolox standard (Sigma-Aldrich, St. Luis, MO, USA) curve (1–2000 μM) for calibration. Results are expressed as μmol Trolox equivalents per g dry weight (μmol TE/g DW).

Total antioxidant capacity (TAC) was measured via the phosphomolybdenum assay [83] and incorporating minor modifications [84]. In this method, 0.05 mL of the methanolic extract was combined with 5 mL of phosphomolybdate reagent and incubated at 95 °C for 60 min. Absorbance of the green PM complex was recorded at 695 nm using a blank for reference. Total antioxidant capacity was calculated from an ascorbic acid standard (Sigma-Aldrich, St. Luis, MO, USA) curve (0–50 mg mL^−1^) and expressed as mg AAE per g dry weight (mg AAE/g DW).

ABTS^•+^ radical scavenging activity was evaluated following the method [85]. The ABTS^•+^ radical cation was generated by mixing 7 mM ABTS^•+^ solution (Sigma-Aldrich, St. Luis, MO, USA) with 2.45 mM potassium persulfate (Merck, Darmstadt, Germany) and allowing the mixture to stand in the dark at room temperature for 12–16 h. Before use, the solution was diluted with methanol to obtain an absorbance of 0.70 ± 0.02 at 734 nm. Then, 0.1 mL of plant extract was added to 3.9 mL of the diluted ABTS^•+^ solution. The decrease in absorbance was measured at 734 nm after 6 min of incubation. A Trolox solution (1–1000 μM) was used as a reference standard (Sigma-Aldrich, St. Luis, MO, USA). Sample absorbance was compared with the Trolox standards, and antioxidant activity was expressed as micromoles of Trolox equivalents per gram of dry weight (μmol TE/g DW).

### 4.4. Measurement of Photosynthesis and Chlorophyll Fluorescence

Gas exchange and chlorophyll fluorescence parameters were measured in *R. serbica* and *R. nathaliae* leaves using a CIRAS-4 infrared gas analyzer system (PP Systems, Amesbury, MA, USA), equipped with a broad-leaf cuvette and an integrated light source, enabling precise control of environmental conditions such as light intensity, CO_2_ concentration, temperature, and humidity during gas exchange analysis and also equipped with a Chlorophyll Fluorescence Module (CFM-4) (PP Systems, Amesbury, MA, USA), under standardized conditions and previously established protocols as described by Kastrati et al. [10].

Gas exchange parameters, including CO_2_ assimilation rate or net photosynthesis (*A,* μmol CO_2_ m^−2^ s^−1^), stomatal conductance (*g_s_*, mmol H_2_O m^−2^ s^−1^), and intercellular CO_2_ concentration or CO_2_ concentration in the sub-stomatal cavity (*C_i_*, μmol mol^−1^), were measured on fully expanded leaves of similar age and size from both plant species at each phase of the investigation. The experimental conditions were standardized as follows: light intensity (PAR, Photosynthetically Active Radiation) was set to 200 µmol m^−2^ s^−1^; RGBW settings were 34% red, 33% green, 33% blue, and 0% white; cuvette temperature was maintained at 25 °C; relative air humidity in the leaf chamber was set to 80%; CO_2_ concentration in the leaf chamber was 400 µmol mol^−1^; cuvette flow rate was 300 cc min^−1^; and analyzer flow rate was 100 cc min^−1^.

Chlorophyll fluorescence parameters, including the maximal photochemical efficiency of PSII (F_v_/F_m_), photochemical efficiency in the light-adapted state (F_v_′/F_m_′), efficiency of photochemical energy conversion in PSII under light (Y(II)), non-regulated energy dissipation quantum yield (Y(NO)), and quantum efficiency of regulated non-photochemical quenching (Y(NPQ)), were measured on the same leaves used for gas exchange. Prior to fluorescence measurements, leaves were dark-adapted for 20 min using light-exclusion clips to allow full relaxation of the photosystems. Subsequently, a saturating light pulse (6000 µmol m^−2^ s^−1^, 1 s) was applied to determine maximal fluorescence in both dark- and light-adapted states. Actinic light was then provided at 200 µmol m^−2^ s^−1^ for steady-state fluorescence analysis. All fluorescence parameters were automatically calculated by the CFM-4 module, and nine leaves per species were measured at various time points following rehydration from the freezing-induced desiccation and from the control and used for statistical evaluation.

### 4.5. SDS-PAGE and Western Blot of Total Leaf Proteins

Total leaf proteins were isolated with the sample buffer as previously described [10]. Samples were separated by SDS-PAGE (SE260 Mighty Small II, Hoefer, Holliston, MA, USA) as described [86] with the only difference being that the gels contained 8.7% (*v*/*v*) glycerol. In total, 30 μg of protein was applied per lane. Semi-dry transfer unit TE70X (Hoefer, Holliston, MA, USA) was used to blot the proteins onto a nitrocellulose membrane. The conditions of SDS-PAGE and semi-dry transfer were described [10]. ROTI^®^Mark WESTERN PLUS (Carl Roth GmbH+Co. KG, Karlsruhe, Germany) prestained protein molecular weight marker for SDS-PAGE and detection on Western blot was used. For immunoblot analysis, the following primary antibodies were tested: anti-dehydrin (AS07 206A), anti-ELIP (AS06 147A), anti-Hsp 17.6 (Class I, AS07 254), anti-Hsp 17.7 (Class II, AS07 255) and anti-FAAH (AS16 3972) from Agrisera (Vännäs, Sweden). As secondary antibody, HRP-conjugated goat anti-rabbit antibody (AS09 602, Agrisera) was used. Enhanced chemiluminescence (ECL) was used to record the immunosignals on X-ray Blue films (Carestream Dental LLC, Atlanta, GA, USA). Films were scanned using an Epson Perfection V850 PRO scanner (Seiko Epson Corporation, Suwa, Japan), and band densitometry was performed with Gel-Pro Analyzer software (Media Cybernetics, Rockville, MD, USA).

### 4.6. Statistical Analyses

Data were presented as arithmetic mean and standard error (±SE). All statistical analyses were conducted using the SPSS software, version 25.0 (IBM Corp., Armonk, NY, USA), including Duncan’s Multiple Range Test at a 5% significance level (*p* ≤ 0.05). A one-way ANOVA was performed to assess significant differences among time points following rehydration (1, 3, 6, 9, 12, 24, 48, and 72 h, as well as on the 7th day) after freezing-induced desiccation, compared with the control (C).

## 5. Conclusions

This study demonstrates that both *R. serbica* and *R. nathaliae* rapidly restore their water status following freezing stress. Photosynthetic activity recovered slowly during the first hours but increased significantly after one day, becoming fully restored within a week, closely linked to rehydration and stomatal reopening. Despite exhibiting stronger initial damage, *R. nathaliae* recovered PSII efficiency faster than *R. serbica*, suggesting a robust repair capacity likely related to its ecological adaptation. Both species activated photoprotective mechanisms during early recovery, with species-specific differences in energy dissipation strategies. Increased antioxidant activity in both species during the early hours of rehydration protects plants from oxidative damage and promotes photosynthetic recovery, thus ensuring successful revival after freezing-induced desiccation. Rehydration from freezing-induced desiccation of *R. nathaliae* and *R. serbica* is accompanied by enhanced levels of protective proteins. Dehydrins and ELIPs play a role during the entire recovery process, while the cytosolic sHsp Class I and Class II proteins act at the onset of recovery (from 1 to 6 h).

Overall, these findings highlight distinct physiological and biochemical strategies employed by *Ramonda* species to cope with freezing stress, providing valuable insights into the protective mechanisms of recovery in resurrection plants, with potential applications for developing stress-resilient crops.

## Figures and Tables

**Table 1:** Secondary metabolites, antioxidant activity and scavenging capacity in leaves of *R. serbica* (*RS*) and *R. nathaliae* (*RN*) during freezing stress (0 h), and at various time points following rehydration from freezing-induced desiccation (1, 3, 6, 9, 12, 24, 48, and 78 h, and 7 days), as well as in control plants (C). The values are presented as mean ± SE, *n* = 9. Identical letters within a graph indicate no significant differences, as determined by Duncan’s Multiple Range Test at the 5% significance level (*p* ≤ 0.05) following ANOVA analysis.

		Time of Rehydration										
Parameters	Species	0 h	1 h	3 h	6 h	9 h	12 h	24 h	48 h	72 h	7 d	C
TP (mg GAE/g DW)	RS	67.85 ^B^ ± 0.49	75.92 ^A^ ± 0.89	76.24 ^A^ ± 1.40	52.43 ^C^ ± 1.55	53.70 ^C^ ± 1.40	52.22 ^C^ ± 1.79	46.26 ^D^ ± 1.30	44.83 ^D^ ± 2.37	33.77 ^E^ ± 0.39	30.87 ^E^ ± 0.36	24.93 ^F^ ± 0.76
	RN	59.41 ^B^ ± 5.34	89.44 ^A^ ± 0.45	90.68 ^A^ ± 0.29	56.71 ^B^ ± 2.29	55.74 ^B^ ± 1.63	48.85 ^C^ ± 1.74	46.54 ^CD^ ± 0.65	41.23 ^D^ ± 0.23	33.42 ^E^ ± 0.70	30.85 E^F^ ± 0.49	26.26 ^F^ ± 1.04
TF (mg CE/g DW)	RS	68.76 ^B^ ± 0.32	73.48 ^A^ ± 1.12	73.25 ^A^ ± 1.22	56.91 ^C^ ± 0.77	46.31 ^D^ ± 0.65	31.84 ^EF^ ± 0.85	31.54 ^EF^ ± 0.74	33.57 ^E^ ± 0.55	29.46 ^F^ ± 0.62	25.71 ^G^ ± 0.35	21.97 ^H^ ± 0.89
	RN	70.10 ^B^ ± 0.94	78.45 ^A^ ± 0.65	78.47 ^A^ ± 0.46	54.37 ^C^ ± 1.14	47.36 ^D^ ± 1.16	37.41 ^E^ ± 0.91	32.88 ^F^ ± 0.38	34.35 ^F^ ± 0.55	25.54 ^G^ ± 0.51	23.44 ^G^ ± 0.68	18.67 ^H^ ± 0.32
FRAP (μmol AAE/g DW)	RS	1331.66 ^C^ ± 31.05	1557.72 ^A^ ± 4.41	1504.72 ^B^ ± 3.86	985.77 ^D^ ± 2.95	577.74 ^E^ ± 2.84	380.79 ^F^ ± 7.48	337.48 ^G^ ± 2.11	280.14 ^H^ ± 1.70	244.62 ^I^ ± 0.69	176.44 ^J^ ± 0.83	145.71 ^K^ ± 0.89
	RN	1844.82 ^B^ ± 14.50	1967.62 ^A^ ± 13.70	1873.26 ^B^ ± 15.84	1529.88 ^C^ ± 15.17	1203.28 ^D^ ± 45.60	747.74 ^E^ ± 4.47	450.89 ^F^ ± 2.70	338.88 ^G^ ± 1.36	283.07 ^H^ ± 0.82	186.97 ^I^ ± 1.25	155.27 ^I^ ± 0.45
DPPH (μmol TE/g DW)	RS	883.05 ^B^ ± 6.25	940.27 ^A^ ± 3.23	747.07 ^C^ ± 8.43	552.36 ^D^ ± 1.38	388.77 ^E^ ± 4.97	335.09 ^F^ ± 1.47	315.63 ^G^ ± 0.64	285.49 ^H^ ± 1.45	225.09 ^I^ ± 0.72	186.62 ^J^ ± 1.33	155.81 ^K^ ± 0.86
	RN	757.54 ^B^ ± 7.48	904.68 ^A^ ± 19.18	724.83 ^C^ ± 2.98	604.82 ^D^ ± 20.48	345.99 ^E^ ± 10.85	334.45 ^EF^ ± 2.24	334.42 ^EF^ ± 2.59	311.62 ^F^ ± 0.42	276.03 ^G^ ± 0.78	242.51 ^H^ ± 0.66	203.38 ^I^ ± 1.18
TAC (mg AAE/g DW)	RS	77.06 ^B^ ± 0.57	80.94 ^A^ ± 2.06	73.27 ^C^ ± 0.75	67.13 ^D^ ± 1.19	50.83 ^E^ ± 0.30	45.68 ^F^ ± 1.09	34.39 ^G^ ± 0.39	32.50 ^GH^ ± 0.59	30.71 ^H^ ± 0.29	27.93 ^I^ ± 0.30	21.45 ^J^ ± 0.59
	RN	117.93 ^A^ ± 1.08	64.34 ^B^ ± 1.11	63.47 ^B^ ± 2.40	46.22 ^C^ ± 3.74	44.57 ^CD^ ± 1.51	41.38 ^DE^ ± 0.63	38.20 ^EF^ ± 0.23	36.07 ^FG^ ± 0.24	33.14 ^GH^ ± 1.27	29.23 ^HI^ ± 0.27	28.26 ^I^ ± 0.19
ABTS^•+^ (μmol TE/g DW)	RS	1006.75 ^C^ ± 8.81	1158.60 ^A^ ± 12.86	1094.87 ^B^ ± 6.25	735.77 ^D^ ± 2.49	648.80 ^E^ ± 2.37	452.17 ^F^ ± 3.82	386.57 ^G^ ± 1.12	316.68 ^H^ ± 2.13	264.36 ^I^ ± 1.35	158.57 ^J^ ± 0.53	155.39 ^J^ ± 0.56
	RN	1258.73 ^B^ ± 3.96	1434.72 ^A^ ± 32.22	1232.13 ^B^ ± 28.02	895.73 ^C^ ± 35.76	564.35 ^D^ ± 16.65	336.86 ^E^ ± 1.44	302.87 ^EF^ ± 0.44	253.93 ^F^ ± 1.29	190.75 ^G^ ± 0.91	155.26 ^G^ ± 0.52	148.27 ^G^ ± 1.63

TP (Total phenols), TF (Total flavonoids), FRAP (Ferric Reducing Antioxidant Power Assay), DPPH (Radical Scavenging Assay), TAC (Determination of Total Antioxidant Capacity), ABTS^•+^ (Radical Scavenging Assay).

## Data Availability

All datasets are contained within the article.
